# Antimicrobial peptide β-defensin-1 expression is upregulated in Alzheimer’s brain

**DOI:** 10.1186/1742-2094-10-127

**Published:** 2013-10-18

**Authors:** Wesley M Williams, Sandy Torres, Sandra L Siedlak, Rudy J Castellani, George Perry, Mark A Smith, Xiongwei Zhu

**Affiliations:** 1 Department of Biological Sciences, School of Dental Medicine, Case Western Reserve University, 2124 Cornell Rd., Cleveland, OH 44106, USA; 2 Department of Pathology, School of Medicine, Case Western Reserve University, 10900 Euclid Ave., Cleveland, OH 44106, USA; 3 Department of Pathology, University of Maryland, 655 W. Baltimore St., Baltimore, MD 21201, USA; 4 UTSA Neurosciences Institute and Department of Biology, University of Texas at San Antonio, 1 UTSA Circle, San Antonio, TX 78249, USA

**Keywords:** Alzheimer’s, Antimicrobial, Brain, Choroid plexus, Defensin, Granulovacuolar, Immunomodulatory, Inflammation

## Abstract

**Background:**

The human β-defensins (hBDs) are a highly conserved family of cationic antimicrobial and immunomodulatory peptides expressed primarily by epithelial cells in response to invasion by bacteria, fungi and some viruses. To date, the most studied members of this family of peptides are hBD-1, -2, and -3. Expression of hBD-1 and -2 has been demonstrated previously in cultured microglia and astrocytes of both mouse and human brain. Unlike inducible hBD-2 and -3, hBD-1 is constitutively expressed and is not generally upregulated by proinflammatory factors. In this study, we investigated whether hBDs, as active components of the innate immune response, are affected by pathological events in the Alzheimer’s disease (AD) brain. We assessed the expression of hBD-1, -2, and -3 in tissue obtained at autopsy from AD and age-matched control brains.

**Methods:**

Fixed and frozen choroid plexus and the CA1 region of the hippocampus were obtained at autopsy from individuals diagnosed with AD, or from age-matched control brains without diagnosed neurodegenerative disease. Histopathologically diagnosed AD brain tissue was obtained for our study. Immunocytochemical analysis was performed using affinity purified polyclonal antibodies directed against hBD-1, -2, or -3. TaqMan gene expression assays were used to quantify the mRNA of hBD-1, -2, and -3 in the choroid plexus and hippocampus. Immunocytochemical detection of iron deposits was achieved using a modified Perl’s stain for redox-active iron. *In vitro* experiments were performed on human primary oral epithelial cells to model the human choroid plexus epithelial response to ferric chloride. Cells were then exposed to ferric chloride added to selected wells at 0, 1, or 10 mM concentrations for 24 h at 37°C. Total mRNA was isolated to quantify hBD-1 mRNA expression by RTqPCR.

**Results:**

hBD-1 peptide is apparent in astrocytes of the AD hippocampus and hippocampal neurons, notably within granulovacuolar degeneration structures (GVD). A higher level of hBD-1 was also seen in the choroid plexus of AD brain in comparison to age-matched control tissue. Increased expression of hBD-1 mRNA was observed only in the choroid plexus of the AD brain when compared to expression level in age-matched control brain. Redox-active iron was also elevated in the AD choroid plexus and *in vitro* addition of Fe^+3^Cl_3_ to cultured epithelial cells induced hBD-1 mRNA expression.

**Conclusions:**

Our findings suggest interplay between hBD-1 and neuroimmunological responses in AD, marked by microglial and astrocytic activation, and increased expression of the peptide within the choroid plexus and accumulation within GVD. As a constitutively expressed component of the innate immune system, we propose that hBD-1 may be of considerable importance early in the disease process. We also demonstrate that increased iron deposition in AD may contribute to the elevated expression of hBD-1 within the choroid plexus. These findings represent a potentially important etiological aspect of Alzheimer’s disease neuropathology not previously reported.

## Background

Mammalian β-defensins are an evolutionarily conserved group of cationic, non-glycosylated antimicrobial and immunomodulatory peptides that are expressed in all mammals, including humans [1,2]. Constitutively expressed human β-defensin-1 and inducible β-defensin-2 (hBD-1 and -2, respectively) are commonly expressed by epithelial cells, and are important contributors to innate host defense [3,4]. These peptides have the potential to act as activators and modulators of adaptive immunity within astrocytes and microglia, brain cells critical to the cerebral neuroinflammatory response, as both cell types have been shown to express hBD-1 and -2 *in vitro*[5-9]. These findings were confirmed by Nakayama et al. who also reported constitutive expression of hBD-1 in the human choroid plexus (CP) [10]. The CP, composed principally of epithelium, endothelium and stroma, is strategically located between the peripheral circulation and cerebral ventricles, and actively participates in regulation of reciprocal immune functions between the brain and peripheral circulation. Direct involvement of the CP in Alzheimer’s disease (AD) neuropathology is well documented with overt epithelial cell atrophy and thickening of epithelial basement membrane with concomitant impairment of synthetic, secretory, and transporter functions, including reduced clearance of amyloid-β [11-14]. In addition to secretion of cerebrospinal fluid (CSF), which is reduced in AD [15], the CP acts as a primary immunosurveillance and defense mechanism against invading pathogens and systemic diseases [16]. The CP is a principal regulator of the intracerebral immune response and inflammation, as indicated by the presence of mast cells, macrophages, granulocytes, and immunocompetent cells within the tissue [17,18]. Other immune alterations specific to AD, including pseudolinear deposits of IgG and deposits of C1q associated with IgM, suggestive of an autoimmune origin have been reported [19]. The cause or causes underlying pathological changes and dysfunction within the CP remain equivocal, although it is apparent that oxidative stress-induced modification of proteins, unrelated to presence of fibrillar amyloid-β, contributes to CP pathology in AD. These modifications include glycation end-products carboxymethyl-lysine and N-carboxyethyl-lysine within the CP of AD brain [20]. The studies cited above suggest CP dysfunction is a likely contributor to neuropathological and inflammatory sequelae in the AD brain.

In this study, we evaluated the expression of hBDs within the AD brain and accumulation of redox-active iron within the CP epithelium. We show that the expression of hBD-1 mRNA is significantly elevated in the Alzheimer’s choroid plexus and the protein is apparent in neurons of the hippocampus. Further, granulovacuolar degeneration (GVD) is found to be a specific site of hBD-1 peptide localization. GVD are small cytoplasmic granules that are increased in neurons of the AD brain; proteins previously described in GVD represent the autophagic ubiquitin-proteasome system, unfolded protein response, endosomal and lysosomal proteins, and tau and amyloid processing events, as well as stress-activated and mitogen-activated protein induction [21-27]. The GVD may play a role similar to that of stress granules, structures that form following various stressors including heat shock, oxidative stress, and, of relevance to this study, infection [28]. The present study provides evidence for GVD formation reflecting a neuroprotective response and suggests that redox-active iron may contribute to alteration of the inflammatory process by upregulating expression of hBD-1, a principal component of the innate host defense system.

In AD, morphological changes and increased immunological responses in the CP contribute to decreased CSF synthesis and secretion, and increased oxidative stress [29]. Despite highly controlled homeostatic iron levels within the healthy brain [30], redox-active iron accumulates in the AD brain [31,32] with a concentration appearing to correlate with the severity of cognitive deficit [33]. Our findings suggest that elevated iron levels in the AD CP, and perhaps also within hippocampal regions, contribute to the increase in hBD-1 expression and an altered innate immune response.

## Methods

### Materials

Brain tissue histopathologically diagnosed with AD using multiple criteria ('definite’ AD by CERAD criteria [34], Braak Stage V-VI [35], and 'high likelihood AD’ by NIA-Reagan criteria [36]), as well as age-matched control brain tissue, were used for our study. Specifically, fixed and frozen CP from AD patients (n = 8) and age-matched controls (n = 7) was obtained from the NICHD Brain and Tissue Bank for Developmental Disorders at the University of Maryland, Baltimore, MD, USA. All tissue samples were obtained within 2 to 12 hours postmortem with similar ages between groups ranging from 66 to 89 years of age. Additionally, fixed and frozen samples of hippocampus were obtained from Case Western Reserve University and University Hospitals of Cleveland, according to approved Institutional Review Board protocols. Samples from control cases (n = 8, ages 74–91) and neuropathologically confirmed AD cases (n = 11, ages 72–94) were used for PCR experiments. For immunocytochemistry, AD cases (n = 9, ages 59–91, mean = 80.1 years) and control (n = 5, ages 53–101, mean = 72.2 years) were analyzed. Skin samples from 3 cases were also collected post-mortem for use as a positive control tissue. Fixed tissues were embedded in paraffin and used for immunocytochemical analysis. Antigen affinity purified polyclonal antibodies directed against hBD-1 (Cat # P25350), -2 (Cat # P161G50), and -3 (Cat # P24150) were purchased from PeproTech (Rocky Hill, NJ, USA). These primary antibodies were produced from rabbit (hBD-1 and -3) or goat sera (hBD-2) pre-immunized with >98% pure recombinant defensin peptide. Antibodies were subsequently purified by affinity chromatography using the respective immobilized defensin peptide matrix (PeproTech). TaqMan gene expression assays were purchased from Applied Biosystems (Life Technologies, Carlsbad, CA, USA).

### RTqPCR

Total RNA was extracted from frozen brain tissue by homogenization in TRI reagent (Ambion, Life Technologies, Carlsbad, CA, USA) and further purified using an RNeasy Mini Kit from Qiagen (Valencia, CA, USA), as instructed by the manufacturer. The purified RNA was treated with Turbo DNase (Ambion) to remove trace DNA. RNA was converted to cDNA using the RETROScript RT-qPCR kit (Ambion), according to the manufacturer’s instructions. Briefly, the purified total RNA (100 ng) was reverse transcribed with cloned Moloney murine leukemia virus reverse transcriptase (5 U) by incubating at 44°C for 60 min, followed by heating at 92°C for 10 min. RT-qPCR using TaqMan gene specific assays with context sequence (GGCCTCAGGTGGTAACTTTCTCACA) was performed in triplicate on cDNA samples in 96-well optical plates on an ABI StepOne Plus Thermocycler (Applied Biosystems). For each 20 μL TaqMan reaction, 2 μL cDNA was mixed with 10 μL 2× TaqMan Fast Universal PCR Master Mix (Applied Biosystems) and 1 μL of 20× TaqMan Assay Mix. Standard fast PCR parameters were used.

Data was normalized to glyceraldehyde dehydrogenase (GAPDH) and shown as the relative RQ value. This stable reference gene was selected by testing AD and age-matched control brain samples on a TaqMan Human Endogenous Control Plate (Applied Biosystems® MicroAmp® Life Technologies, Carlsbad, CA, USA). The data indicated five stable genes that could be used for normalization. Using the geNORM (PrimerDesign) algorithm, the best choice for normalization of RT-qPCR data from AD vs. age-matched control brain samples was a combination of two genes, human acidic ribosomal protein (huPO) and human β_2_-microglobulin (huβ2m). Stability of huGAPDH was second only to that of huβ_2_m/huPO out of a total of 11 suggested reference genes.

### Immunocytochemical analysis

Paraffin was removed from tissue sections with two changes of xylene; tissue sections were then hydrated through descending concentrations of ethanol to Tris buffered saline (TBS, 50 mM Tris, 150 mM NaCl, pH = 7.6). Endogenous peroxidase activity was ablated by 30 min incubation in 3% hydrogen peroxide in methanol. Antigen retrieval using citrate buffer (pH 6) in a pressure cooker (Biocare Medical, Concord, CA, USA) was performed. Non-specific antibody binding sites were blocked with a 30 minute incubation in 10% normal goat serum (NGS) in TBS, and sections incubated overnight in primary antibody diluted in 1% NGS in TBS at 4°C. Secondary antibodies were applied followed by PAP complex and staining developed with 3’-3’-diaminobenzidine (Dako North America, Carpenteria, CA, USA). Sections were dehydrated and mounted with Permount mounting medium.

To verify the presence of hBD-1 peptide in glial cells, double label immunocytochemistry was performed on hippocampal sections from either AD or non-AD brain using rabbit polyclonal antibody to hBD-1 (PeproTech) detected using the PAP method with brown reaction product DAB and with mouse monoclonal antibody to glial fibrillary acidic protein (GFAP, clone GA5, Millipore), detected with the alkaline phosphatase anti-alkaline phosphatase method using Fast Blue as chromogen. Stained sections were mounted with Crystalmount, coverslipped and imaged using Zeiss Axiocam mounted on a Zeiss Axiophot (Carl Zeiss Microscopy, LLC, Thornwood, NY, USA). Epidermal sections of human skin known to express hBD-1 were also subjected to hBD-1 antibody as a positive control.

Immunocytochemical detection of iron deposits within the CP and hippocampus was achieved by a modified Perl’s stain for redox-active iron using 7% potassium ferrocyanide in 3% HCl [30,31]. The CP epithelial cell layer was identified using an affinity purified rabbit polyclonal antibody (ABBiotec, San Diago, CA, USA) to transthyretin, a marker for CP epithelial cells.

### Cell culture of human primary epithelial cells and exposure to redox-active iron

Primary oral epithelial cells were used to model the response of CP epithelial cells to redox-active iron (Fe^+3^). Cells were obtained from human donors according to the policies described by the Institutional Review Board at Case Western Reserve University. After informed consent by selected patients oral epithelial cells were extracted from healthy oral tissue overlying impacted third molars, as described by Oda and Watson [37]. In brief, cells were cultured in EpiLife growth medium (Cascade Biologists, Portland, OR, USA) and maintained under 5% CO_2_ at 37°C. Primary cells were grown as a monolayer under serum-free conditions. Upon reaching confluence, cells from at least three donors were trypsinized, detached, and pooled before reseeding in EpiLife growth medium at 4 × 10^4^ cells/well in 6-well culture plates. Before challenging with dilutions of ferric chloride, cells were incubated under 5% CO_2_ at 37°C, and cultured until ~80–95% confluence (~3 × 10^5^ cells/well). Ferric chloride at 1 and 10 mM was added to selected wells and cells were incubated for an additional 24 h. Experiments were terminated by aspiration of the incubation medium and addition of TRIzol reagent (Life Technologies, Grand Island, NY, USA) to each well for subsequent quantitation of hBD-1 mRNA by RT-qPCR.

### Statistical analysis

Data are presented as the Mean ± S.E. Statistical analysis was performed using exact probabilities for the non-parametric Mann Whitney 'U’ test where N_1_ = 7, N_2_ = 8 (CP), or N_1_ = 8, N_2_ = 11 (hippocampus).

## Results

### Beta defensin-1 peptide is increased in hippocampus and CP of the AD brain

**Figure 1:**
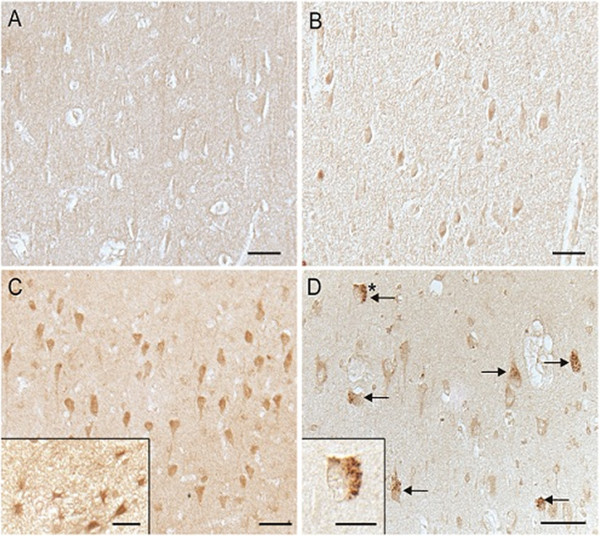
**Immunocytochemical demonstration of hBD-1 in the hippocampus.** Age-matched controls **(A,B)** vs. AD **(C,D)** shows an elevated protein expression in the cytoplasm of pyramidal neurons in AD compared to non-AD control brain. The expected localization of hBD-1 in glial cells was also demonstrated in AD cases **(C**, inset**)**. In AD cases, GVD is also specifically stained **(D**, arrows**)**. In **D**, inset shows a higher magnification image of the GVD-containing neuron marked with an asterisk (*). Scale bar **A–D** = 50 μm; Insets **C,D** = 20 μm.

Examination of hippocampal sections revealed the apparent accumulation of hBD-1 peptide in the cytoplasm of pyramidal neurons in only 1 out of 5 age-matched control cases (Figure 1A,B) versus 8 out of 9 AD cases (Figure 1C,D). The hBD-1 peptide was also tentatively identified in astrocytes of the AD brain (Figure 1C, inset), an expected finding since hBD-1 expression has been reported in cultured human astrocytes. Most striking was the strong and specific staining of GVD within pyramidal neurons of AD hippocampus (Figure 1D arrows, and inset).

**Figure 2:**
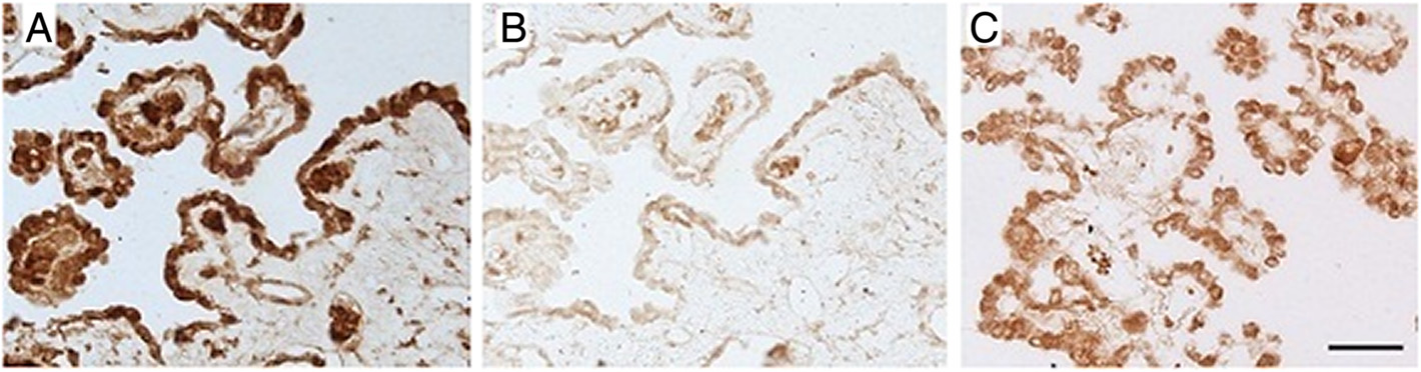
**Transthyretin is a marker protein for epithelial cells.** Epithelial cells of the choroid plexus (CP) are demonstrated in an age-matched control brain using antibody to transthyretin **(A)**. While hBD-1 is only weakly expressed in control CP epithelium **(B)**, the peptide is strongly expressed by these cells in the AD CP **(C)**.

The CP epithelial cell layer from age-matched control brain was clearly delineated by the presence of transthyretin (Figure 2A). Within the CP, the hBD-1 peptide was detected only in epithelial cells, consistent with presence of intracellular hBD-1. We noted weak expression of the peptide in non-AD age-matched CP (Figure 2B), but a strong, distinct signal in the CP epithelium from AD brain (Figure 2C). We could not detect hBD-2 or -3 peptide in either CP or hippocampal tissue sections derived from either AD or control brain (data not shown).

**Figure 3:**
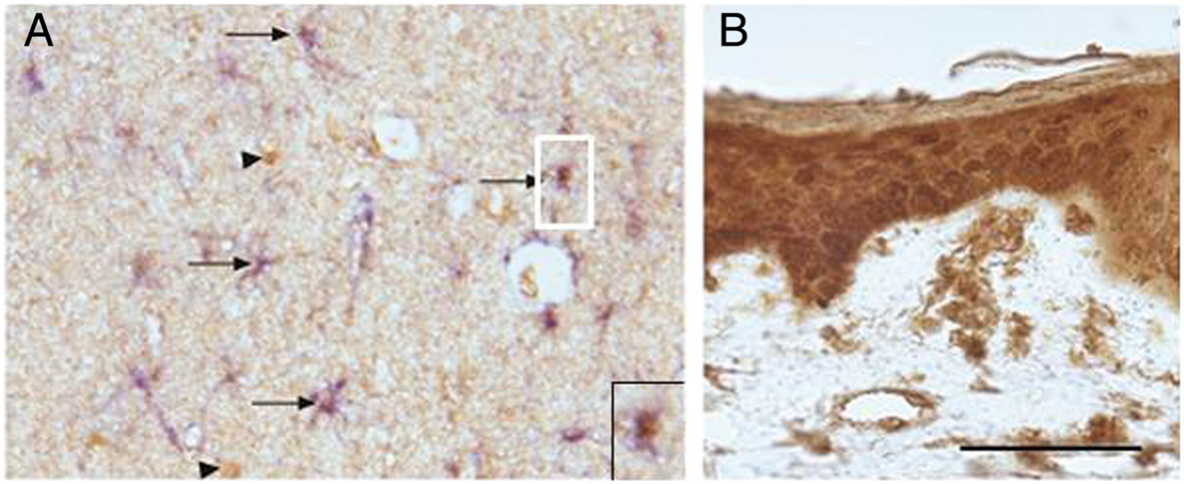
**hBD-1 is apparent in astroglial cells of the hippocampus.** The expected localization of hBD-1 brown reaction product in glial cells was demonstrated in AD cases by colocalization with an antibody to GFAP (blue) **(A**, inset**)**. Arrows mark the numerous glial cells containing both GFAP and hBD-1, while arrowheads denote neurons containing only the brown hBD-1 reaction product. Specificity of the antibody and method used for detection of hBD-1 was tested on tissue sections of human epidermal skin cells, known to express hBD-1, as a positive control **(B)**. Scale bar (inset) = 50 μm.

In Figure 3A we show the presence of hBD-1 peptide in glial cells expressing GFAP, a glial cell marker. Human skin used as a positive control for the presence of hBD-1 shows high expression of the peptide in the upper epidermal layer (Figure 3B).

### Expression of hBD-1 mRNA is elevated in CP epithelium, but not in hippocampal cells of the AD brain

**Figure 4:**
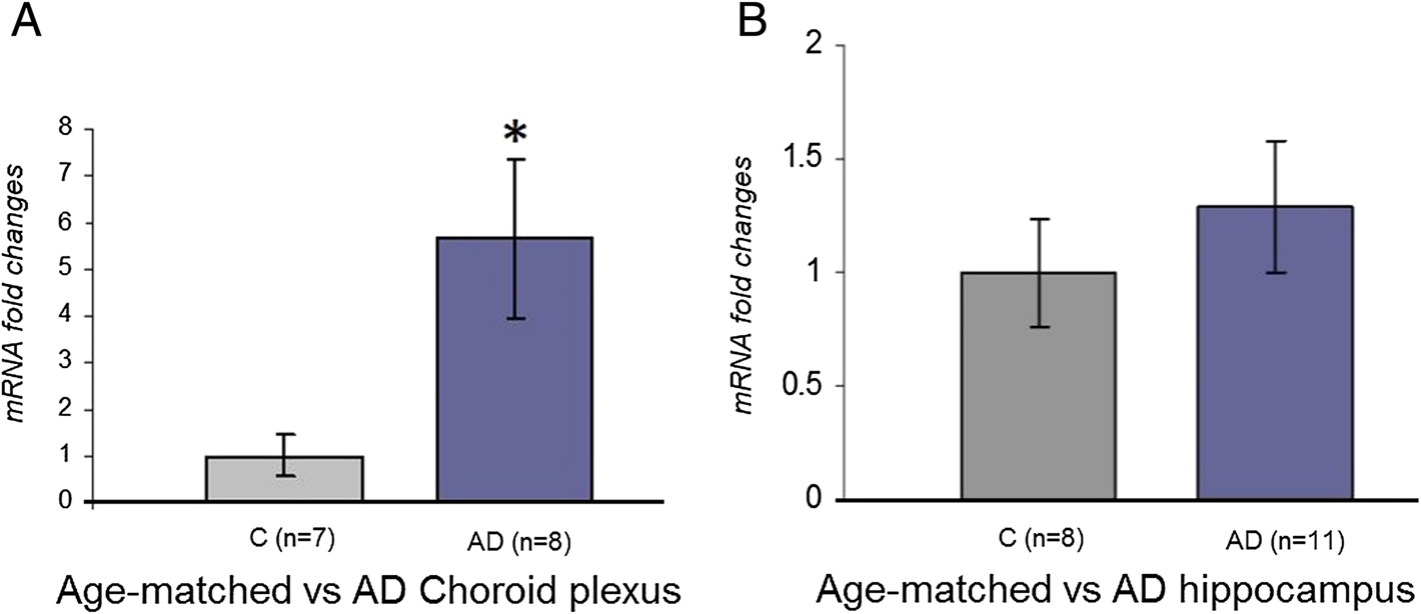
**hBD-1 expression is increased in the AD CP epithelium.** hBD-1 mRNA expression in the CP epithelium from AD brain (n = 8) shows a statistically significant (*P = 0.02) elevation in peptide expression, compared to age-matched control tissue (n = 7) **(A)**. Results are expressed as the mean ± S.E. No statistically significant difference in hBD-1 mRNA expression was found between the hippocampus from AD brain (n = 11) compared to age-matched controls (n = 8) **(B)**.

Using RT-qPCR we assessed the expression of hBD-1, -2, and -3 mRNAs in both the hippocampus and CP from AD brains and age-matched controls. Expression of hBD-1 mRNA is significantly elevated in the AD CP in comparison to age-matched controls (*P = 0.02, Figure 4A). However, the levels of hBD-1 mRNA were similar in both AD and control hippocampus (Figure 4B). Expression of mRNA for hBD-2 and -3 was not detected in either CP or hippocampus from AD or control brains (data not shown).

### Redox-active iron deposition occurs in CP epithelial cells of the AD brain

**Figure 5:**
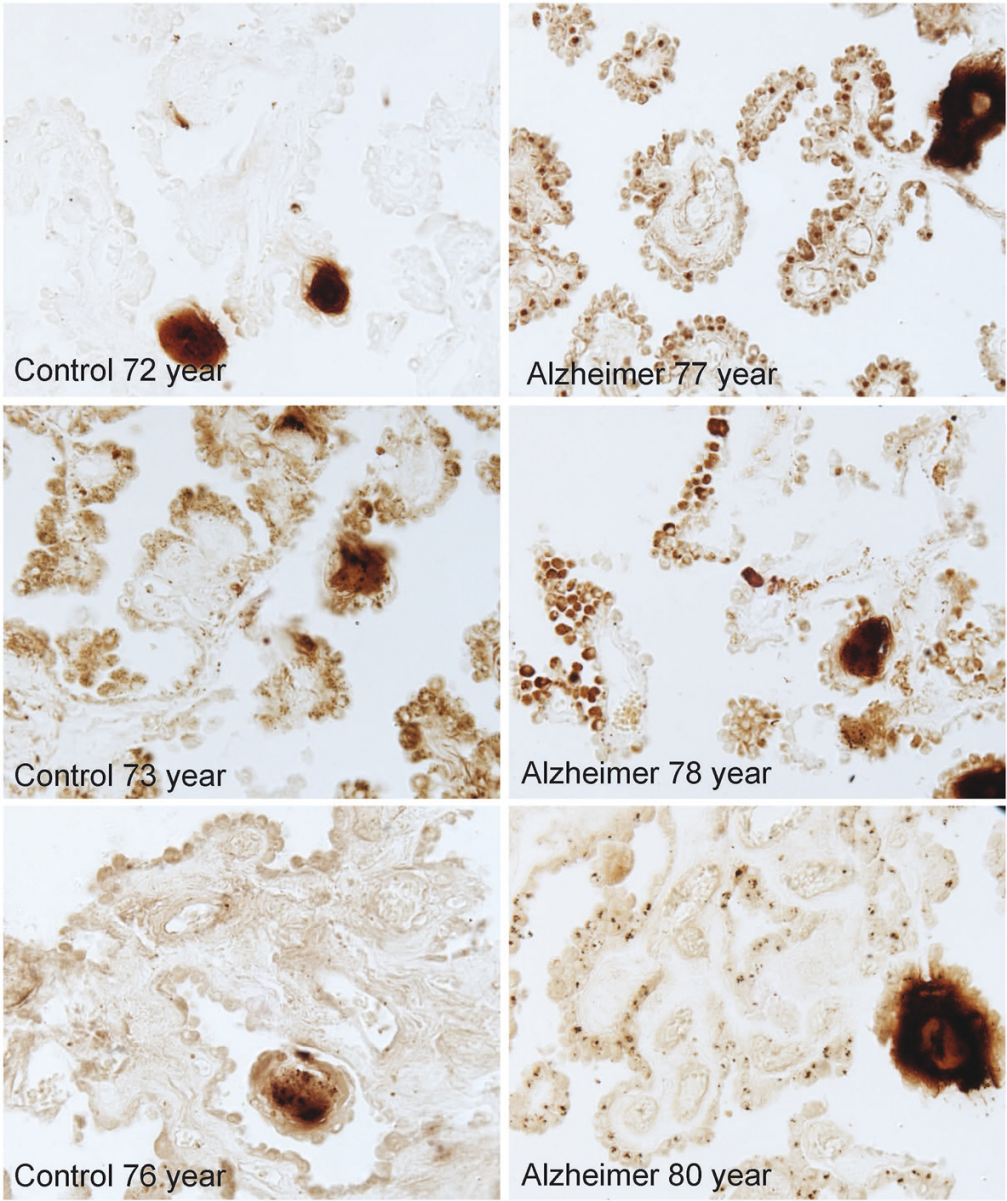
**Immunocytochemical demonstration of redox-active iron deposits within the CP epithelium of the AD brain.** Redox-active iron deposits are not detectable in the CP of age-matched control brain. Redox-active iron was detected using a modified Perl’s stain [30].

Using a modification of the Perl’s stain to specifically detect Fe^+3^, a redox-active form of non-heme iron, we visualized a strong signal within the CP epithelium of the AD cases, but not in epithelium of the control CP (Figure 5).

### Redox-active iron upregulates *in vitro* expression of hBD-1 in human epithelial cells

**Figure 6:**
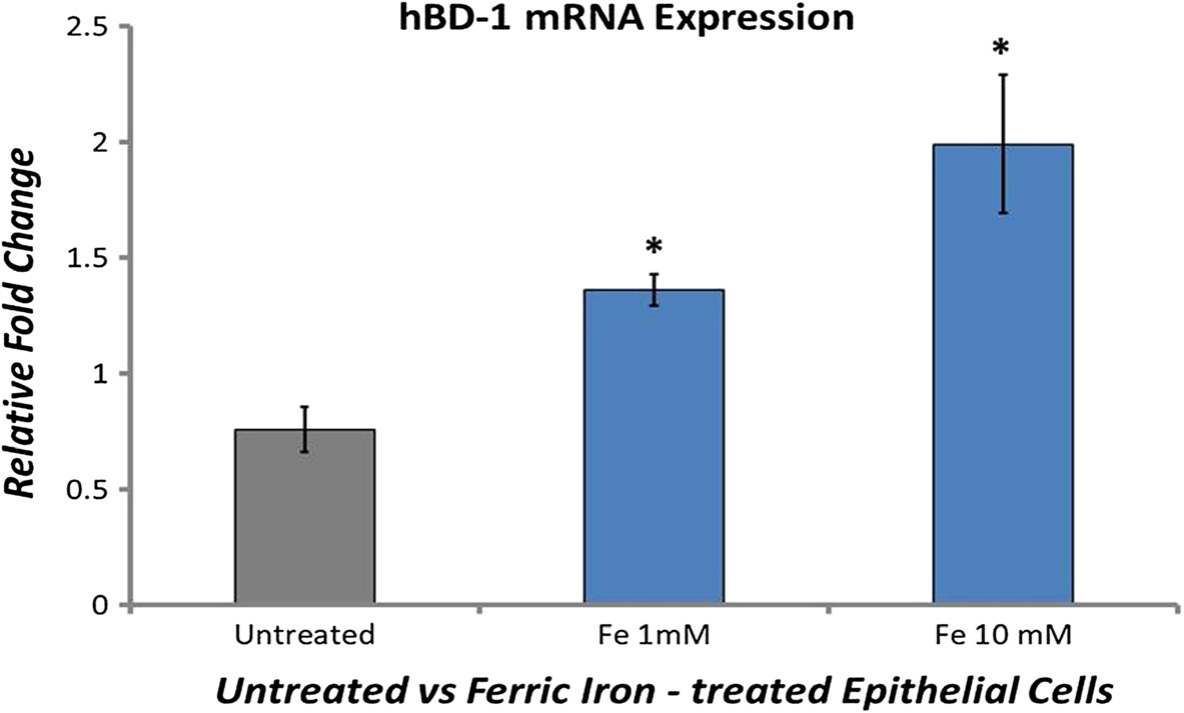
**Redox-active iron increases hBD-1 expression in epithelial cells *in vitro*.** hBD-1 mRNA expression in human epithelium grown in culture subjected to sham-exposure or exposed to 1 or 10 mM Fe^+3^Cl_3_ during a 24 h incubation at 37°C. Results are expressed as the mean ± S.D. of 3 independent experiments (*P = 0.014).

The 24 h exposure of cultured human primary epithelial cells to either 1 or 10 mM Fe^+3^Cl_3_ induced a detectable dose-dependent upregulation of hBD-1 mRNA (n = 3, U = 0, *P* = 0.014) relative to untreated control cells (Figure 6).

## Discussion

The hBD gene cluster is located in chromosome 8p22-23, a region containing multiple genes related to innate immunity and the nervous system [38]. With the exception of constitutively expressed hBD-1, inducible hBD-2 and -3 are generally upregulated by an inflammatory environment [39,40]. Thus, surprisingly, we detected the upregulated expression of hBD-1, which is generally unresponsive to inflammation. Conversely, we failed to detect expression of hBD-2 or hBD-3, despite the reported presence of neuroinflammation in affected regions of the AD brain, including the hippocampus and CP [41,42]. The hBD-1 gene (DEFB1) is a single copy gene with several SNPs that have been associated with the pathogenesis of some chronic inflammatory diseases, including asthma and chronic obstructive pulmonary disease [43,44]. Genomic variations in DEFB1 also contribute to the clinical course of severe sepsis and inflammation with presence of specific haplotypes associated with either increased susceptibility to, or protection from, severe infection and fatal outcome [45]. This latter study underscores a possible role for hBD-1 in modulating inflammation within the CNS and suggests that the observed elevation in hBD-1 expression within the AD brain is a protective response to an inflammatory environment. One obvious question is whether susceptibility to inflammation within the CNS is influenced by specific genomic variants of DEFB1, and whether individuals with AD express a gene variant that is less effective in modulating inflammation.

hBD-1 is the only known hBD that is constitutively expressed and is rarely upregulated [46-48]. The peptide is not only immunomodulatory through chemotaxis of memory T lymphocytes and stimulation to maturity of immature myeloid dendritic cells [49,50], but responsive and cytotoxic to gram-negative bacteria and adenovirus. Expression of hBD-1 is not significantly affected by exposure to LPS, *Salmonella* Dublin, or *Escherichia coli*[51]. This study is the first to report the effect of a neurodegenerative disease on expression of a well characterized antimicrobial and immunomodulatory peptide. Here, we show significant elevation of the hBD-1 peptide and mRNA level in CP epithelium, and increased peptide in hippocampal neurons of the AD brain relative to age-matched control brain tissues.

The underlying cause(s) of increased hBD-1 expression in the AD brain is not apparent. It is also unclear whether the hBD-1 peptide could be functioning as an antimicrobial, immunomodulator or both, and whether functionality of the peptide affects clinical symptoms of AD. We thus propose several mechanisms that might explain our results based upon our own recent findings, and the published studies of others.

The presence of hBD-1 within the CP, an interface between brain parenchyma and the systemic circulation, suggests that increased expression in the AD brain CP could be a response to microbial-derived stimuli impinging from the systemic side of the blood-CSF barrier. Influenza, Sendai and Herpes simplex viruses are known to modulate hBD-1 expression [52], and Herpes simplex DNA has been shown to localize quite specifically to the amyloid plaques of the AD brain [53]. To date though, identification of pathogenic determinants in AD, including bacteria, viruses and fungi has been an inconsistent finding, and no single pathogenic agent has been categorically identified as a contributor to AD neuropathology or cognitive decline [54]. Nonetheless, the functional role that hBD-1 plays within the CP could be influenced by pathogenic stimuli, such as non-self-pattern recognition associated with AD.

Another intriguing mechanism that could underlie changes in hBD-1 expression within the CNS is a non-inflammatory upregulation of the peptide by altered biological clock control of peptide expression through c-myc. Sherman et al. [55] have shown that albumin can upregulate expression of hBD-1 in HCT-116 cells concomitant with elevation of c-myc, a response that was inhibited by addition of a c-myc inhibitor. Additional studies showed that the 5 kbp promoter region of the defensin peptide contains putative binding sites (E-box-like sequences) for c-myc that are binding sites for the dimerized transcription factors CLOCK and BMAL1 [55,56]; noteworthy is that components of the innate immune system are regulated by the biological clock [57,58]. Thus, hBD-1 expression level may conform to influence from the biological clock and binding of c-myc to the defensin promoter region [51]. This mechanism may be quite relevant to hBD expression in AD in view of the altered circadian rhythm of the master clock cells in the superchiasmatic nucleus of the AD brain [59]. Although alterations in biological clock function occur in both aging and AD, the circadian disturbance appears to be more pronounced in the AD brain [60].

It is possible that the observed increase in hBD-1 within the CP and hippocampus occurs by different mechanisms. We believe that the increased presence of hBD-1 within neurons may represent an acute neuroprotective response of the brain to extra- or intra-cerebral factors. Presence of hBD-1 within GVD, structures which can function as autophagosomes [61] but also show both morphological and protein-based similarities to stress granules [28], may signal a neuronal response to induction of cellular stress by a chronic, proinflammatory environment. Since the majority of neurons containing GVD are not yet undergoing neurodegeneration, i.e., they lack hyperphosphorylated tau, neurofibrillary pathology, and are not apoptotic, the apparent elevation of hBD-1 in these structures could help identify the cellular response to stress. Additional study of mild cognitive impairment, including cases with mild AD, is necessary to clarify the role of glial activation known to occur early on in the disease process [62].

An increase in hBD-1 peptide in the hippocampus of the AD brain without a respective increase in mRNA would not be without precedent. In fact, attempts to correlate protein levels with similar changes in mRNA expression have met with mixed results [63]. The increase in hBD-1 peptide in the AD hippocampus may reflect upregulation of a post-translational pathway such as that described by Acquaviva et al. [64] to explain their observed increase in the frataxin protein without an associated increase in expressed mRNA. The lack of an increase in mRNA expression, corresponding to an increase in protein level is not an infrequent occurrence as is demonstrated by a study on new molecular targets for the anticancer drug, rapamycin [65]. In this study, out of 56 yeast proteins increased in abundance following exposure to rapamycin, 26 did so without a detectable change in mRNA expression. It is also possible that accumulation of the hBD-1 peptide in the GVD may occur over years and derive from glial cell death and release of hBD-1 peptide that subsequently is sequestered via the hypothesized protective function of neuronal GVD. Borenstein et al. [66] proposed a similar hypothesis to explain the presence of defensin peptides (NP-1, -2, and -5) in infected rabbit testis. These defensins are expressed by macrophages and neutrophils, but both these cell-types were absent from the tissue samples in which the defensins were detected. Similarly, Sahasrabudhe et al. [67] noted increased expression of hBD-1 in inflamed human salivary gland ducts, but without a corresponding increase in hBD-1 mRNA. In their study, detectable levels of the peptide were demonstrated by immunoblot. Unfortunately, we could not verify the presence of this small (3.9 kDa) peptide by Western blot of hippocampal tissue. We attribute this to a peptide concentration in the brain that is below the level of detectability for our antibody. It cannot be completely rules out that our antibody is cross-reacting with another protein; however, we believe this to be unlikely, given that there is no cross-reactivity between our antibody to hBD-1 and other beta-defensins having close homology to hBD-1 (PeproTech, personal communication). Nevertheless, without quantitative verification that our antibody is, in fact, detecting hBD-1 in the hippocampus we make the tentative assertion that hBD-1 is present and its expression upregulated in the AD hippocampus.

Redox-active iron insidiously accumulates within the ageing brain, but the rate of accumulation is accelerated in several neurodegenerative diseases, including AD [68,69]. Accumulation occurs primarily in regions of the brain that show susceptibility to AD neuropathology [70]. Of particular interest is the underlying cause of hBD-1 upregulation within the epithelium of the AD CP where we also observed increased sequestering of redox-active iron. We are unaware of any histological verification of abnormal iron accumulation specifically within the CP epithelium. Iron levels within the CNS are largely regulated by the blood-CSF and blood–brain barriers [71,72]. Dysregulation of iron homeostasis with resulting increase of redox-active iron is a probable contributor to AD neuropathology and inflammation through the promotion of oxidative conditions [73]. We propose that the elevated level of redox-active iron within the CP epithelium underlies our observed increase in hBD-1. This proposal is supported by the demonstrated role of iron as a regulator of immune function and our findings from the present study showing that iron can induce the upregulation of hBD-1 in cultured epithelium (Figure 6). Furthermore, Lee et al. [74] have shown that both cellular iron deficiency and overload can modulate elements of the innate and adaptive immune response, including the upregulated expression of the chemotactic cytokine MIP3α/CCL20. As a constitutively expressed antimicrobial/immunomodulatory peptide, hBD-1 expression would be expected to respond to signals impinging upon the epithelium indicative of pathogen infection. Our demonstrated ability to upregulate hBD-1 mRNA expression *in vitro* through exposure of epithelial cells to redox-active ferric iron (Fe^+3^Cl_3_) but not ferrous iron (data not shown), suggests that redox-active iron underlies upregulated expression of hBD-1, perhaps through activation of cellular pathways and innate immune defenses normally upregulated by intracellular invasion of iron-containing pathogens [75]. In fact, both iron depletion and iron overload have been shown to alter patterns of cellular gene expression [76]. As an interface between the systemic circulation and CSF, the CP epithelium undoubtedly plays a critical role in regulating iron metabolism and therefore iron availability to pathogens attempting to invade the CNS [77]. We suggest that the increased presence of intracellular iron within the CP, regardless of the underlying cause, contributes to the upregulation of the hBD-1 peptide and possibly other components of the innate immune response by the CP epithelium.

Modulation of the innate and adaptive immune response in the CNS is an intricate process likely involving the hBD-1 peptide. Recently, Cribbs et al. [78] showed extensive upregulation of innate immune system pathways in post mortem hippocampus and cortex from aged and AD-afflicted individuals relative to these same regions from young (20 to 59 years) cases. Our observed increase in hBD-1, a component of the innate immune system, within the AD brain is therefore consistent with this observed activation of innate immune pathways and underscores the need for further study of hBD-1 function and possible contribution of genomic variation in the DEFB1 gene to neuroinflammation in AD. Absence of detectable hBD-2 or -3 in either AD or age-matched control brains suggests hBD-1 may have a unique influence on innate immune response within the brain. However, future studies will require additional comparisons to young adult brain in order to accurately assess hBD function.

The possibility that hBD-1 may function as an antimicrobial or immuno-modulating element in neuroinflammation is supported by the literature cited above and by the novel findings presented in this study. The role or roles that specific antimicrobial peptides, such as hBD-1, may play in neurodegenerative diseases, including AD, requires further investigation in order to clarify their contributions to the overall host immune response in these diseases.

## Conclusions

Our study results suggest the expression of the human antimicrobial peptide hBD-1 is elevated in the CP and hippocampus of the AD brain, relative to hBD-1 levels in the brain of non-demented, age-matched controls. This increased expression localized to the CP, an interface between the brain and systemic circulation, and the apparent increase in the neuronal cell bodies and glia within the hippocampus, suggest an active role for hBD-1 as a potential modulator of the host innate immune response within the CNS.

## Abbreviations

CP: Choroid plexus; CSF: Cerebrospinal fluid; DEFB1: Human beta-defensin 1 gene; GAPDH: Glyceraldehyde 3-phosphate dehydrogenase; GVD: Granulovacuolar degeneration; hBDs: Human beta-defensins; huβ2m: Human β2-microglobulin; huPO: Human acidic ribosomal protein.

## Competing interests

All authors declare that they have no competing interests.

## Authors’ contributions

WW proposed the original hypothesis, wrote the manuscript, performed RNA extractions, and data analyses; ST performed RTqPCR, and contributed to writing the manuscript; RC obtained and contributed post-mortem tissues for analysis; SS performed immunocytochemical analyses, and contributed to writing the manuscript; MS, GP, and XZ contributed their considerable expertise to directing the course of the study. All authors read and approved the final version of the manuscript.
